# Reproductive and Cardiometabolic Characterization of a Letrozole- and High-Fat Diet-Induced PMOS-like Rat Model: An Experimental Study

**DOI:** 10.3390/pathophysiology33030061

**Published:** 2026-08-11

**Authors:** Milica Milinkovic Sorgic, Aleksandar Matic, Vladimir Jakovljevic, Nikola Jovic, Sladjana Novakovic, Teodora Todorovic, Jovan Milosavljevic, Bozidar Pindovic, Jasmina Sretenovic, Petar Canovic, Jovana Jakovljevic Uzelac, Jovana Joksimovic Jovic

**Affiliations:** 1 Department of Biochemistry, Faculty of Medical Sciences, University of Kragujevac, Svetozara Markovica 69, 34000 Kragujevac, Serbia; milicamilinkovic15@yahoo.com (M.M.S.); petar.c89@gmail.com (P.C.); 2 Center of Excellence for Redox Balance Research in Cardiovascular and Metabolic Disorders, 34000 Kragujevac, Serbia; drvladakgbg@yahoo.com (V.J.); pindovic.bozidar@gmail.com (B.P.); drj.sretenovic@gmail.com (J.S.); 3 Department of Surgery, Faculty of Medical Sciences, University of Kragujevac, Svetozara Markovica 69, 34000 Kragujevac, Serbia; 4 University Clinical Center Kragujevac, 34000 Kragujevac, Serbia; docctorny@gmail.com; 5 Department of Physiology, Faculty of Medical Sciences, University of Kragujevac, Svetozara Markovica 69, 34000 Kragujevac, Serbia; jovan.milosavljevic1997@gmail.com; 6 Department of Human Pathology, I.M. Sechenov First Moscow State Medical University, Moscow 119048, Russia; 7 Department of Gynecology and Obstetrics, Faculty of Medical Sciences, University of Kragujevac, Svetozara Markovica 69, 34000 Kragujevac, Serbia; 8 Faculty of Medical Sciences, University of Kragujevac, 34000 Kragujevac, Serbia; sladja.novakovic.sumarice@gmail.com; 9 Department of Pharmacy, Faculty of Medical Sciences, University of Kragujevac, 34000 Kragujevac, Serbia; teodora.anicic95@gmail.com; 10 Institute of Medical Physiology “Richard Burian”, Faculty of Medicine, University of Belgrade, 11000 Belgrade, Serbia; jovanavjakovljevic@gmail.com

**Keywords:** polycystic ovary syndrome, reproduction, oxidative stress, vaginal cytology, morphometric analysis, ovary, uterus, heart

## Abstract

Background/Objectives: Polyendocrine metabolic ovarian syndrome (PMOS) is a complex endocrine–metabolic disorder of multifactorial etiology, driven by interactions among hyperandrogenism, metabolic dysfunction, oxidative stress, and chronic low-grade inflammation that interact to promote reproductive dysfunction and increase risk of cardiometabolic complications. Although the combination of letrozole and high-fat diet (LET + HFD) represents a widely used experimental approach for inducing a PMOS-like phenotype in rats, its multisystem pathophysiological features remain incompletely characterized. The present study aimed to characterize reproductive, cardiometabolic, hormonal, inflammatory, oxidative, and morphometric alterations associated with a LET + HFD-induced PMOS-like phenotype in rats. Methods: PMOS-like model was induced in rats over a 21-day period of orally administered letrozole combined with a high-fat diet. Results: Body weight gain was higher, while uterine weight was lower in the PMOS group compared with controls. Metabolic parameters suggested insulin resistance, while hormonal profiling revealed increased total testosterone and LH levels, accompanied by reduced FSH, estradiol, and progesterone levels. Elevated triglycerides and reduced HDL levels were also observed in the PMOS group. Morphometric analysis revealed numerous atretic and large thin-walled cystic follicles in the ovaries, accompanied by thinning of the uterine luminal and glandular epithelium, stromal layer, and hypoplasia of endometrial glands. Both the longitudinal diameter and cross-sectional area of cardiomyocytes were increased in the PMOS group, together with nuclear hypertrophy and enhanced myocardial collagen deposition. In addition, altered oxidative stress markers, including increased lipid peroxidation and reduced glutathione levels, were observed, whereas IL-6, IL-1β, and IL-23 were not significantly altered. Conclusions: The LET + HFD model reproduces key reproductive and cardiometabolic features of PMOS, extending beyond reproductive dysfunction to involve oxidative and structural alterations in multiple organs. These findings support its use for investigating PMOS pathophysiology and evaluating potential preventive and therapeutic strategies.

## 1. Introduction

Polyendocrine metabolic ovarian syndrome (PMOS, formerly known as polycystic ovary syndrome, PCOS) is one of the most common endocrine–metabolic disorders affecting women of reproductive age, with an estimated global prevalence of up to 20%, while nearly 70% of affected women remain undiagnosed [1,2]. It is characterized by reproductive dysfunction, hyperandrogenism, and polycystic ovarian morphology [3,4]. Beyond these reproductive abnormalities, it is increasingly recognized as a multisystem cardiometabolic disorder [5] associated with obesity, insulin resistance, dyslipidemia, type 2 diabetes [6,7], hypertension [7,8], endothelial dysfunction with altered nitric oxide bioavailability [9] and increased cardiovascular risk.

Current understanding of PMOS pathophysiology indicates that the syndrome is characterized by dysregulation of the hypothalamic–pituitary–ovarian axis, leading to excessive LH secretion, relative FSH deficiency, ovarian hyperandrogenism, disrupted folliculogenesis, and anovulation. In addition, oxidative stress and chronic low-grade inflammation are increasingly recognized as important contributors to both reproductive and metabolic dysfunction, promoting endocrine imbalance and cardiometabolic complications [10]. Notably, hyperandrogenism and insulin resistance are considered major drivers of the increased cardiovascular risk associated with PMOS, contributing to endothelial dysfunction, oxidative stress, adverse lipid profiles, and visceral adiposity. These interconnected mechanisms promote early cardiometabolic alterations and support the concept of PMOS as a multisystem cardiometabolic disorder rather than solely a reproductive disease [11]. A better understanding of these complex interactions requires well-characterized experimental models that faithfully reproduce both the reproductive and cardiometabolic features of PMOS.

Animal models are indispensable for investigating PMOS pathophysiology and evaluating novel therapeutic strategies. Among the available rodent models, the combination of letrozole, a nonsteroidal aromatase inhibitor, and a high-fat diet (LET + HFD) has emerged as one of the most extensively validated and widely adopted experimental approaches because it reproducibly induces both reproductive and metabolic characteristics of a PMOS-like phenotype more consistently than letrozole alone [12]. The LET + HFD protocol has been successfully employed in numerous experimental studies using a relatively short induction period, demonstrating reproducible endocrine, metabolic, and ovarian alterations while providing a practical platform for mechanistic and therapeutic investigations. The combined protocol induces hyperandrogenism, disrupted estrous cyclicity, insulin resistance, obesity, and dyslipidemia, thereby closely resembling the endocrine–metabolic phenotype observed in many women with PMOS [12,13].

Although the LET + HFD model has been widely used to evaluate therapeutic interventions and explore individual pathogenic mechanisms, its comprehensive phenotypic characterization remains limited. In particular, reproductive, endocrine, metabolic, oxidative, inflammatory, cardiac, and multi-organ histomorphological changes have rarely been assessed simultaneously within a single experimental study. Therefore, we hypothesized that the LET + HFD-induced PMOS-like phenotype is characterized by multisystem alterations that extend beyond reproductive dysfunction and encompass early cardiometabolic remodeling.

Therefore, the present study aimed to comprehensively characterize the LET + HFD-induced PMOS-like phenotype in female rats through integrated endocrine, metabolic, inflammatory, oxidative, histological, and morphometric assessments across multiple organ systems. In addition to defining the reproductive phenotype, we investigated myocardial collagen deposition and structural cardiac remodeling, providing, to the best of our knowledge, the first evidence of these cardiac alterations in this widely used model and extending its pathophysiological characterization beyond the reproductive system.

## 2. Materials and Methods

### 2.1. Ethical Approval

All animal procedures were approved by the Ethical Committee for Experimental Animal Welfare, Faculty of Medical Sciences, University of Kragujevac, Serbia (approval No. 01-2260/1; 18 March 2024), and were conducted in accordance with the European Council Directive 86/609/EEC, national regulations on laboratory animal welfare, and the ARRIVE guidelines.

### 2.2. Animals

Twelve six-week-old female Wistar albino rats (150–170 g) were obtained from the Department of Laboratory and Experimental Animal Breeding, Military Medical Academy, Belgrade, Serbia. Animals were housed under standard laboratory conditions (23 ± 1 °C; 12 h light/12 h dark cycle, lights on at 08:00 h) with ad libitum access to food and water. Standard polypropylene cages with bedding were used. During a 12-day acclimatization period, estrous cyclicity was monitored daily by vaginal cytology, and only females exhibiting regular 4–5-day estrous cycles were included in the study.

An a priori sample size calculation was performed using G*Power 3.1.9.7 [14]. Based on testosterone data from a previously published study using the same PMOS model [12], the estimated effect size was d = 3.17, indicating a minimum sample size of four animals per group (α = 0.05, power = 0.95). Therefore, six animals per group were included to ensure adequate statistical power while adhering to the 3R principles for the reduction in animal use.

### 2.3. Experimental Design and PMOS Induction

Experimental workflow is presented in Figure S1. Regularly cycling rats were randomly assigned to either the control (CON) or PMOS group (*n* = 6/group) using a computer-generated randomization sequence. PMOS was induced over 21 days by daily oral administration of letrozole (Femara^®^; Novartis Pharma Stein AG, Stein, Switzerland, 1 mg/kg body weight) suspended in 1% carboxymethylcellulose (CMC), combined with a high-fat diet providing approximately 60% of total caloric intake from fat, according to a protocol adopted from previous validated studies demonstrating reliable induction of the PMOS-like phenotype [3,12]. Control animals received standard chow and 1% CMC vehicle. Doses were adjusted according to body weight throughout the study. Body weight was recorded at baseline, twice weekly throughout the study, and immediately before sacrifice.

### 2.4. Estrus Cycle Monitoring

Estrous cycle stage was determined by daily vaginal cytology. Vaginal lavage samples were collected each morning (09:00–10:00 h) using saline, stained with hematoxylin, and examined under a light microscope. Cycle stages were identified according to the predominant cell types as previously described [15]. Estrous cyclicity was monitored daily during the final 12 days of the experimental period, allowing assessment of multiple consecutive cycles. Persistent diestrus, reflecting chronic anovulation and disrupted estrous cyclicity, together with hyperandrogenism and the presence of cystic ovarian follicles, was considered confirmation of successful induction of the PMOS-like phenotype.

### 2.5. Sample Collection

At the end of the experimental period, animals were anesthetized with isoflurane and euthanized by decapitation. Trunk blood was collected into tubes with and without anticoagulant for plasma and serum preparation, respectively. Following centrifugation (3000 rpm), serum, plasma, and erythrocyte lysates were prepared and stored at −20 °C until analysis. The heart, ovaries, a 1 cm segment of the left uterine horn, and periovarian and subcutaneous adipose tissues were collected. The heart, ovaries, and uterine segments were weighed before fixation. Only representative portions of periovarian and subcutaneous adipose tissue were collected for histological analysis; therefore, the weight of the entire adipose depots was not determined. To minimize the influence of physiological hormonal fluctuations, all animals were sacrificed during the diestrus phase.

#### Homogenization of Right Ovary

The right ovaries were dissected, weighed, rinsed with ice-cold phosphate-buffered saline (pH 7.4), and homogenized (1:10, w/v). After centrifugation (10,000 rpm, 10 min, 4 °C), the supernatants were collected and stored at −80 °C until biochemical analysis [16].

### 2.6. Histological and Morphometrical Analysis

After fixation in 4% formaldehyde for 48 h, tissue samples were dehydrated through a graded ethanol series (50%, 70%, 96%, and 100%), cleared in xylene, embedded in paraffin, and sectioned at 4–5 μm using a rotary microtome. Before staining, sections were deparaffinized in xylene, rehydrated through decreasing ethanol concentrations, rinsed in distilled water, and stained with Mayer’s hematoxylin and 2% eosin (H&E). Sections were mounted with DPX medium and coverslipped. Additional 5-μm heart sections were stained with Picrosirius Red for collagen assessment.

#### 2.6.1. Ovarian Histology

Three ovarian sections per animal (the central section and sections located approximately 150 μm anterior and posterior to the center) were examined [17]. The numbers of cystic follicles and corpora lutea were quantified. Cystic follicles were identified according to established morphological criteria, including a thin granulosa cell layer (one to four cell layers), absence of luteinization, and enlarged follicular diameter [18].

#### 2.6.2. Uterine Histology

Three transverse uterine segments from each animal were embedded in the same paraffin block, and one representative section from each segment was analyzed. Three independent measurements of the luminal epithelium, glandular epithelium, stromal layer, and total endometrial thickness were performed in each section [19]. Measurements were obtained perpendicular to the epithelial surface in well-oriented transverse sections while avoiding tissue folds, glandular distortions, and processing artifacts. Mean values were calculated for each section and subsequently averaged to obtain a single value for each animal.

#### 2.6.3. Adipose Tissue Histology

Morphometric analysis of adipocytes was performed on H&E-stained periovarian and subcutaneous adipose tissue sections using ImageJ software (Version 1.54 g) with the Adiposoft plugin [20]. Three non-consecutive sections per animal, separated by approximately 150 μm, were analyzed. Five non-overlapping representative microscopic fields per section were captured using a 20× objective. Following image calibration, adipocytes were identified using the semi-automated Adiposoft detection algorithm. Artifacts, damaged cells, blood vessels, and incorrectly segmented structures were manually excluded. Adipocyte cross-sectional area and equivalent diameter were measured, and mean values from all fields and sections were averaged to obtain a single value for each animal. Photomicrographs for ovaries, uterus and adipose tissue were made using a Leica DM2500 microscope equipped with a Leica Flexacam i5 digital camera (Leica Microsystems, Wetzlar, Germany).

#### 2.6.4. Cardiac Histology

Heart tissue sections stained with H&E were used for morphometric analysis of cardiomyocyte cross-sectional area and longitudinal diameter, whereas Picrosirius Red-stained sections were used for collagen quantification. Images were acquired using an Olympus BX51 microscope equipped with a digital camera. Morphometric analyses were performed according to our previously published protocols [6,21,22,23] using calibrated Axiovision (Zeiss, White Plains, NY, USA) and Image-Pro Plus 7.0 (Media Cybernetics, Rockville, MD, USA) software. Cardiomyocyte cross-sectional area, longitudinal diameter, and collagen deposition were evaluated in 10 representative tissue sections per animal. Longitudinal diameter was measured only in cardiomyocytes with a visible nucleus. Representative myocardial regions excluding large blood vessels, endocardium, and pericardium were selected for analysis. For collagen quantification, regions of interest were defined, irrelevant areas were excluded, and collagen-positive area was determined by automated image segmentation. Results were expressed as the percentage of collagen-positive area relative to the total analyzed tissue area. Collagen quantification was performed independently by two blinded investigators, and the averaged values were used for statistical analysis.

All histological and morphometric analyses were performed by investigators blinded to group allocation.

### 2.7. Biochemical Analysis

#### Metabolic Assessment

Following overnight fasting (12 h), basal blood glucose was measured using a Bayer glucometer from the tail vein, and serum insulin concentrations were determined using a commercial ELISA kit (FineTest, Wuhan, China). The Homeostatic Model Assessment of Insulin Resistance (HOMA-IR) was calculated as fasting insulin (mIU/L) × fasting glucose (mmol/L)/22.5.

### 2.8. Lipid Profile

The concentrations of total cholesterol (TC), triglycerides (TG), and high-density lipoproteins (HDL) were measured using an automated biochemical analyzer (Dimension Xpand, Siemens, IL, USA) with commercially available diagnostic kits provided by Siemens Healthcare Diagnostics (Frimley, Camberley, Surrey, UK). LDL cholesterol was calculated individually for each animal using the Friedewald equation expressed in SI units: LDL-C (mmol/L) = TC − HDL-C − TG/2.2.

### 2.9. Hormonal Assays

Serum concentrations of total testosterone (T), progesterone (P4), estradiol (E2), luteinizing hormone (LH), and follicle-stimulating hormone (FSH) were determined. Testosterone and progesterone were measured using an Elecsys 2010 automated analyzer (Roche Diagnostics, Mannheim, Germany) based on the electrochemiluminescence immunoassay (ECLIA) technique with commercially available Elecsys Testosterone II and Progesterone III assay kits. The analytical sensitivities were 0.025 ng/mL for testosterone and 0.05 ng/mL for progesterone. Estradiol and LH concentrations were determined using commercial ELISA kits (FineTest^®^, Wuhan, Hubei, China; Rat E2 ELISA Kit, ER150; Rat LH ELISA Kit, ER1123), whereas FSH was measured using a Rat FSH ELISA Kit (ELK Biotechnology^®^, Wuhan, Hubei, China; ELK1315), according to the manufacturers’ instructions. Hormone concentrations were calculated from standard curves. Testosterone and progesterone concentrations were expressed in ng/mL, estradiol in pg/mL, and LH and FSH in mIU/mL. The analytical sensitivities of the E2, LH, and FSH assays were 7.5 pg/mL, 0.188 mIU/mL, and 0.71 mIU/mL, respectively.

### 2.10. Oxidative Stress Parameters

Oxidative stress parameters were determined in plasma, erythrocyte lysates, and ovarian homogenates using validated spectrophotometric methods, as previously described [24]. Plasma concentrations of TBARS, NO_2_^−^, H_2_O_2_, and O_2_^−^ were determined as markers of oxidative damage. Activities of SOD and CAT, together with GSH levels, were measured in erythrocyte lysates and ovarian homogenates to evaluate antioxidant defense. Results were expressed in the appropriate units for each parameter.

### 2.11. Determination of Total Protein Content

Total protein concentration in tissue samples was determined using the standard biuret method.

### 2.12. Inflammatory Markers

Serum concentrations of interleukin-6 (IL-6), interleukin-1β (IL-1β), and interleukin-23 (IL-23) were determined by ELISA using commercially available kits (FineTest^®^, Wuhan, China). Cytokine concentrations were expressed in pg/mL. The analytical sensitivities of the IL-6, IL-1β, and IL-23 assays were 37.5, 18.75, and 9.375 pg/mL, respectively.

### 2.13. Statistical Analysis

Statistical analyses were performed using SPSS statistical program version 22.0. Data are presented as mean ± SEM. Normality was assessed using the Shapiro–Wilk test. Variables with a normal distribution were analyzed using the unpaired Student’s t-test, whereas variables that did not satisfy the normality assumption were analyzed using the Mann–Whitney U test (Table S1). A *p* value < 0.05 was considered statistically significant. The individual animal was considered the experimental unit for all statistical analyses. For morphometric assessments, measurements obtained from multiple tissue sections or cells were averaged per animal before group comparisons were performed.

## 3. Results

No abnormal clinical signs, morbidity, or behavioral abnormalities unrelated to the experimental intervention were observed throughout the study. No mortality occurred during the experimental period, and all animals completed the study according to the experimental protocol.

### 3.1. Impact of the LET + HFD on the Body Weight Gain and Estrus Cycle

**Figure 1:**
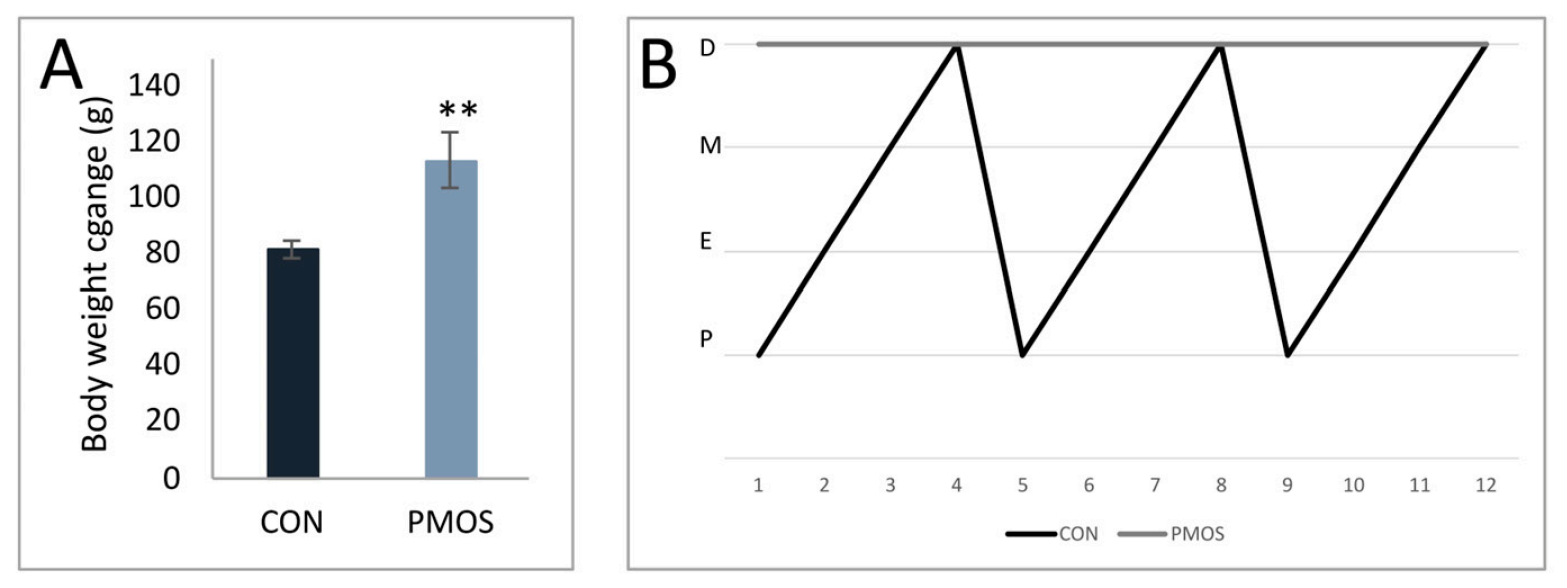
Changes in body weight gain (**A**) and representative estrous cycle patterns observed in the experimental groups (**B**); the cycles shown are typical for control (CON) and LET + HFD-induced PMOS rats, although minor individual variations may occur. Bars represent means ± SEM; ** statistical significance at level *p* < 0.01 compared to the CON group. (*n* = 6 rats per group).

At the onset of the experimental protocol, no statistically significant difference in BW was observed between the CON and PMOS groups. As shown in Figure 1A, a significant change in body weight gain was observed in the PMOS group at the end of experimental protocol in comparison with the age-matched CON (38.47%, *p* < 0.01). Furthermore, estrous cycle analysis (Figure 1B) revealed a persistent diestrus phase in the PMOS group. Estrous cycle pattern of each animal during the final 12 days of the experimental protocol is presented in Table S2.

As shown in Table 1, ovarian, uterine and heart weights were expressed to 100 g of body weight. In PMOS group uterine weight was significantly reduced (*p* < 0.01) compared to CON group. No significant difference was observed in the ovarian and heart weights between the groups.

### 3.2. Impact of LET + HFD on the Alteration of Ovarian, Uterine, Adipose Tissue and Cardiomyocyte Morphology

**Figure 2:**
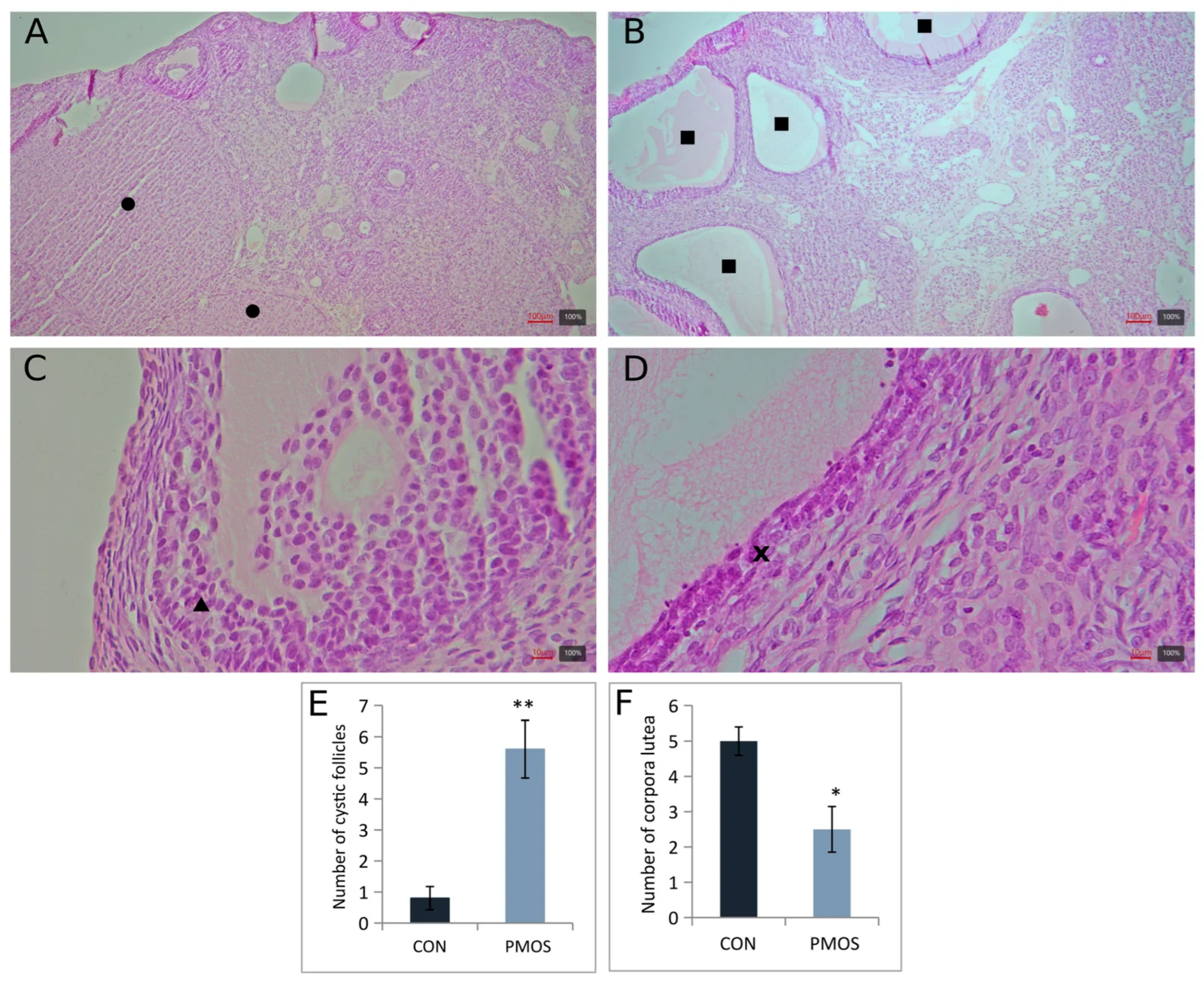
Ovarian histology ((**A**)—CON, (**B**)—PMOS, magnification 5×, scale bar=100 μm; (**C**)—CON, (**D**)—PMOS, magnification 20x, scale bar = 10 μm); Number of ovarian cysts per group (**E**); Number of corpora lutea per group (**F**); ●—corpus luteum; ■—cystally dilated follicles; ▲—thick granulosa layer; **x**—thin granulosa layer; Bars represent means ± SEM; * statistical significance at level *p* < 0.05 compared to the CON group; ** statistical significance at level *p* < 0.01 compared to the CON group. (*n* = 6 rats per group).

As shown in Figure 2A, the ovaries from the CON group exhibited a healthy morphology, containing numerous follicles at various stages of folliculogenesis. In contrast, the PMOS group (Figure 2B) displayed multiple cystically dilated follicles with flattened walls. Figure 2C illustrates a healthy follicle with a thick granulosa layer, representative of the CON group, whereas Figure 2D shows a cystic follicle with a noticeably thinner granulosa layer, typical of the PMOS group. After PMOS modeling, the number of cystic follicles was significantly higher (600%) in the PMOS group compared to the CON group (*p* < 0.01) (Figure 2E), while the number of corpora lutea was significantly lower (50%) (*p* < 0.05) (Figure 2F). Gross figures of ovaries are presented in Figure S2.

**Figure 3:**
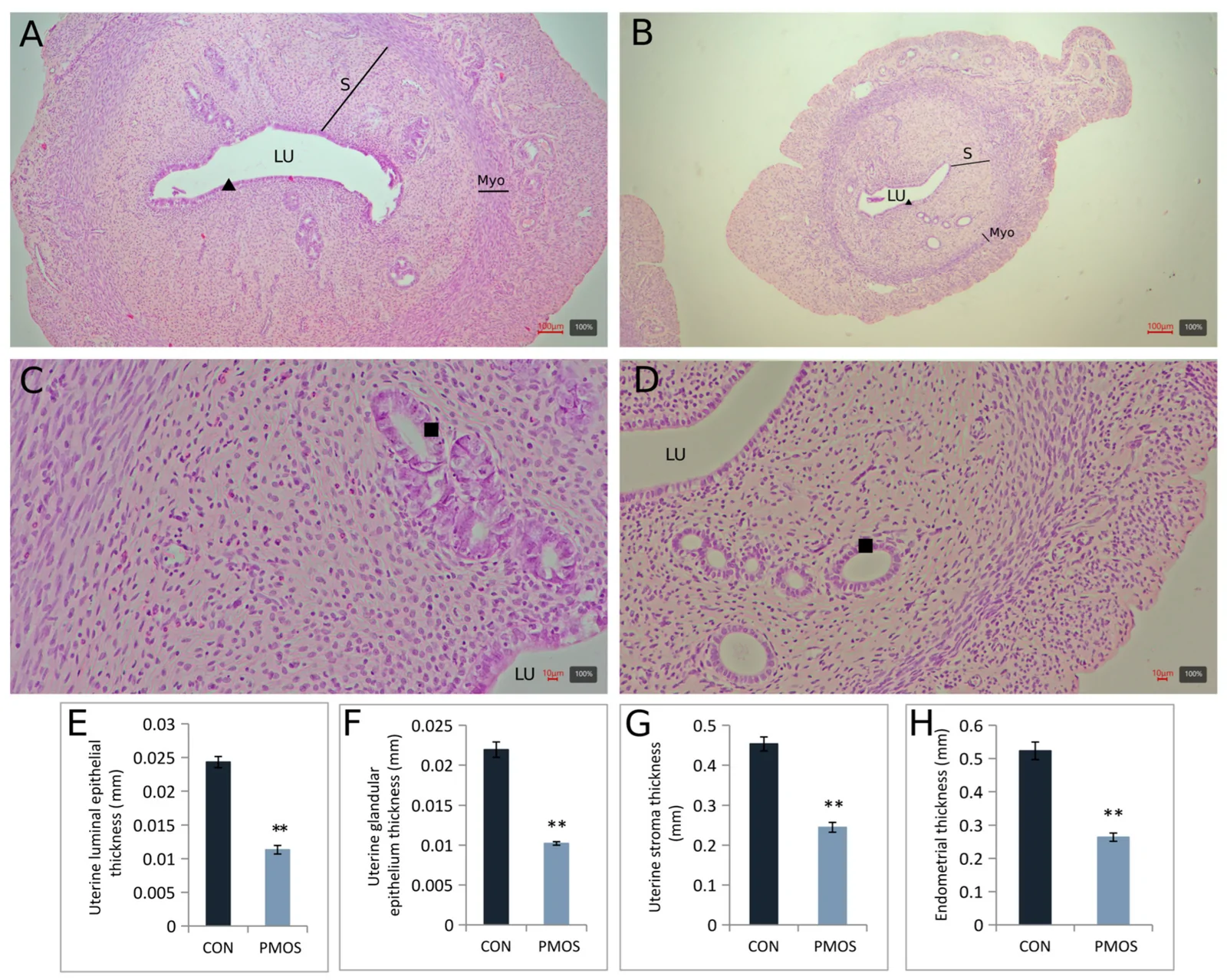
Uterine histology ((**A**)—CON, (**B**)—PMOS, magnification 5×, scale bar = 100 μm; (**C**)—CON, (**D**)—PMOS, magnification 20×, scale bar = 10 μm); uterine luminal epithelium thickness (**E**); uterine glandular epithelium thickness (**F**); uterine stroma thickness (**G**); endometrial thickness (**H**); LU—lumen; S—stroma; Myo—myometrium; ▲—luminal epithelium; ■—glandular epithelium; Bars represent means ± SEM; ** statistical significance at level *p* < 0.01 compared to the CON group. (*n* = 6 rats per group).

As shown in Figure 3A, the uterus from the CON group displayed a healthy morphology with normal size and structure, while the uterus from the PMOS group (Figure 3B) appeared visibly smaller. Figure 3C presents a representative section of a healthy uterus characterized by well-developed glands and a normal luminal epithelium, whereas Figure 3D shows uterine tissue from the PMOS group, characterized by stromal thinning, glandular hypoplasia, and reduced vascularity, accompanied by overall endometrial thinning. Uterine luminal epithelium thickness (Figure 3E), uterine glandular epithelium thickness (Figure 3F), uterine stroma thickness (Figure 3G), and endometrial thickness (Figure 3H) were significantly reduced (53.43%, 53.43%, 45.98% and 49.54%, respectively) in the PMOS group relative to the controls (*p* < 0.01).

**Figure 4:**
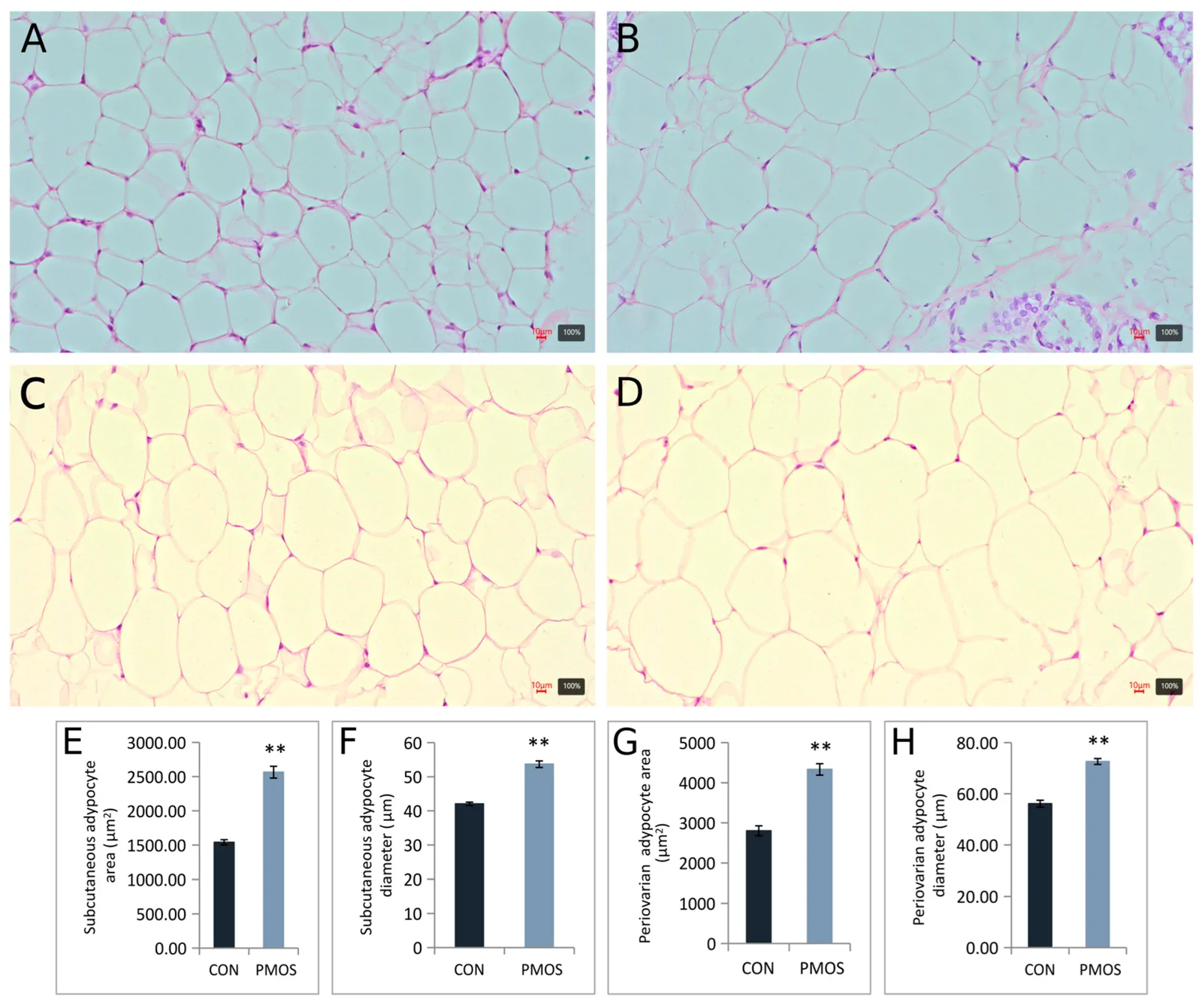
Subcutaneous adipocyte tissue histology ((**A**)—CON, (**B**)—PMOS, magnification 20×, scale bar = 10 μm); periovarian adipocyte tissue histology ((**C**)—CON, (**D**)—PMOS, magnification 20×, scale bar = 10 μm); subcutaneous adipocyte area (**E**); subcutaneous adipocyte diameter (**F**); periovarian adipocyte area (**G**); periovarian adipocyte diameter (**H**); Bars represent means ± SEM; ** statistical significance at level *p* < 0.01 compared to the CON group. (*n* = 6 rats per group).

Representative sections of the animals’ subcutaneous (Figure 4A,B) and periovarian adipose tissue (Figure 4C,D) are shown in Figure 4. In the PMOS group, both the subcutaneous adipocyte area (Figure 4E) and diameter (Figure 4F) were significantly increased (66.42% and 27.68%, respectively). Furthermore, the PMOS group showed a significant enlargement of periovarian adipocyte area (Figure 4H) as well as diameter (54.62% and 29.40%, respectively) (Figure 4G).

**Figure 5:**
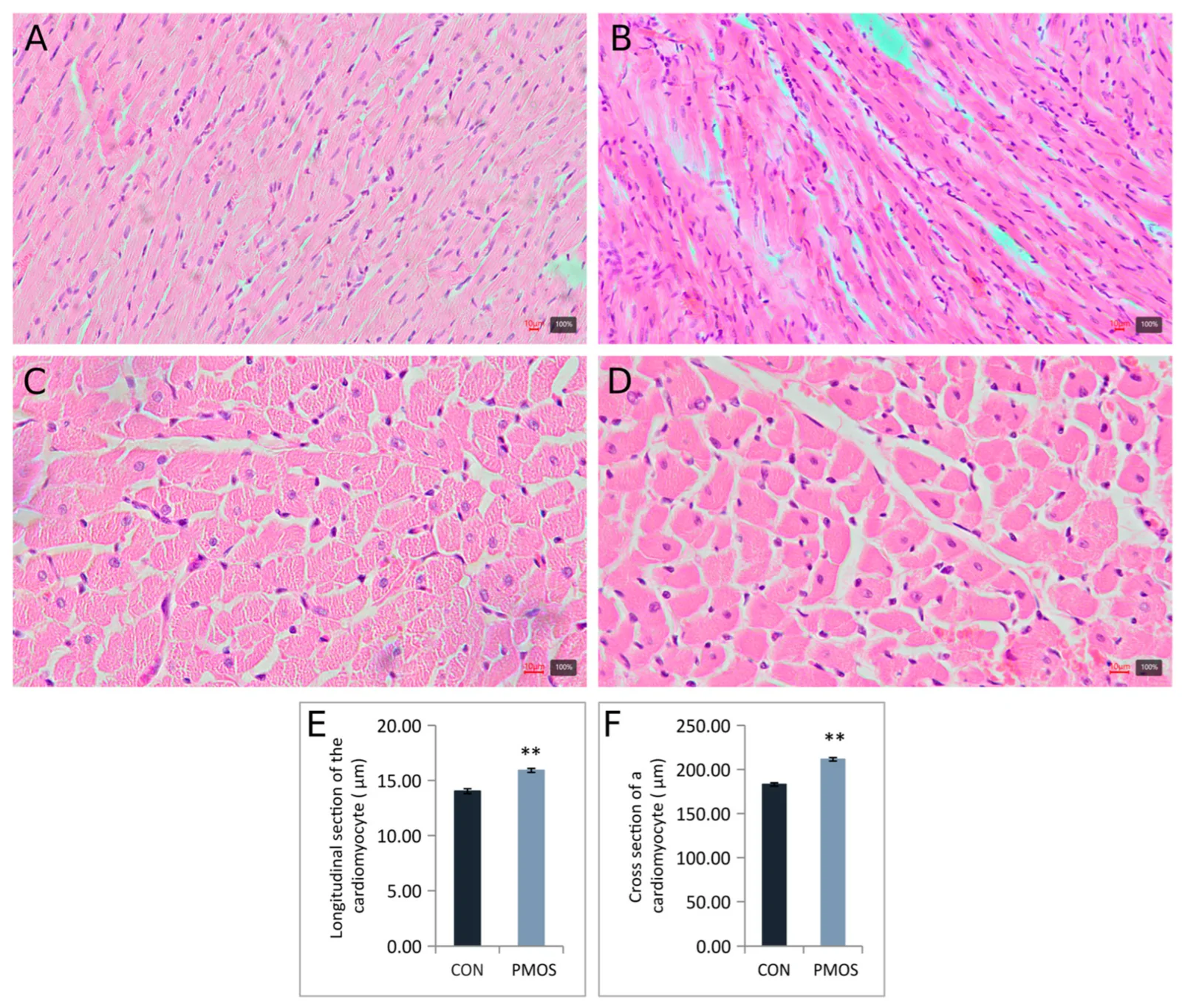
Heart histology ((**A**)—CON, (**B**)—PMOS, longitudinal sections, magnification 20×, scale bar = 10 μm; (**C**)—CON, (**D**)—PMOS, cross-section of cardiomyocytes, magnification 20×, scale bar = 10 μm); Morphometric analysis of longitudinal section of cardiomyocytes (**E**) and cross-section of cardiomyocytes (**F**); Bars represent means ± SEM; ** statistical significance at level *p* < 0.01 compared to the CON group. (*n* = 6 rats per group).

Figure 5 presents representative photomicrographs of cardiomyocyte sections from the animals. In the CON group, a regular arrangement of muscle fibers was observed, with no evidence of hypercellularity or cardiomyocyte hypertrophy (Figure 5A,C). In the PMOS group, morphometric changes consistent with cardiomyocyte hypertrophy-like alterations were evident (Figure 5B,D). Both the longitudinal diameter (Figure 5E) and cross-sectional area (Figure 5F) of cardiomyocytes showed significantly higher values (13.42% and 15.54%, respectively) in the PMOS group compared to the CON group.

**Figure 6:**
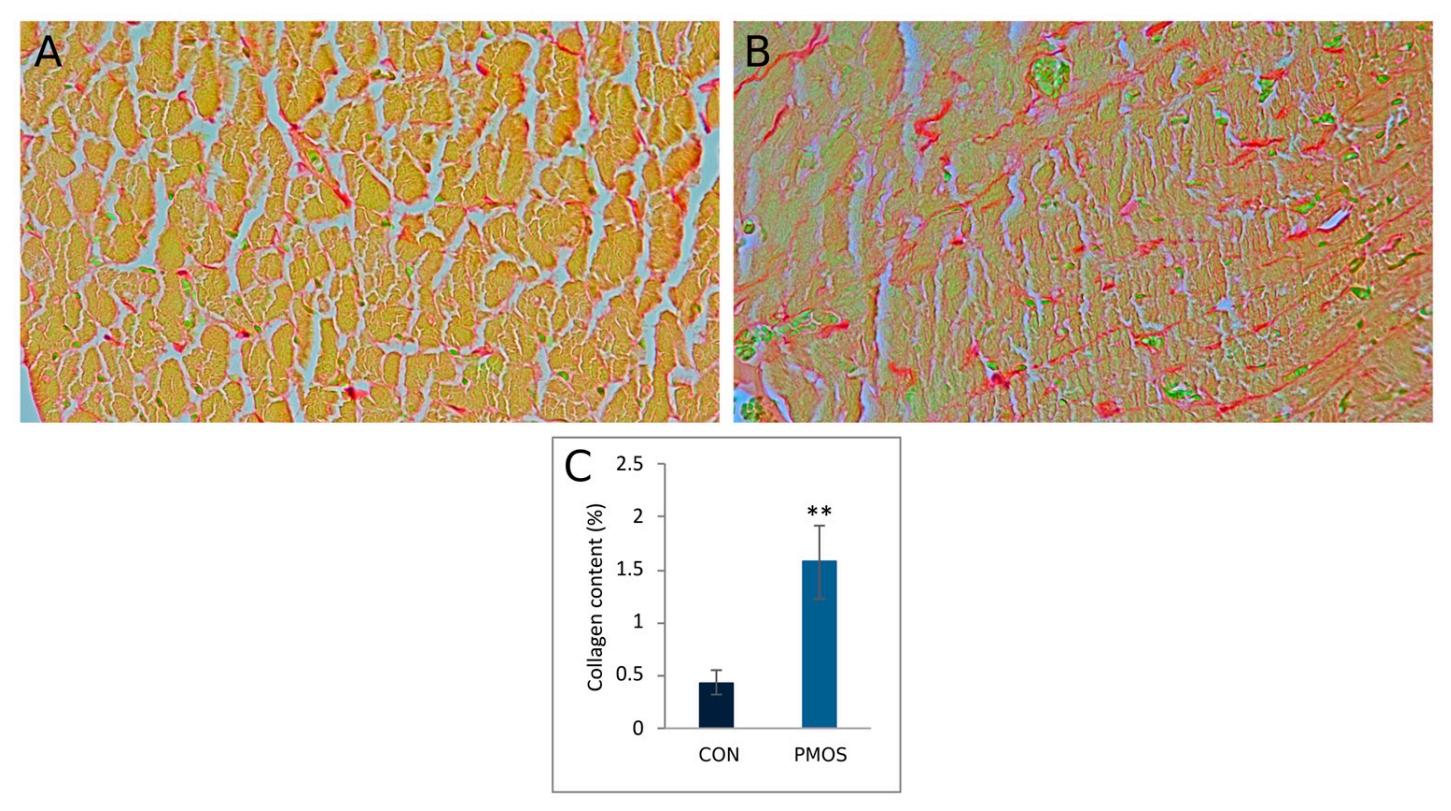
Picrosirius Red-stained myocardial sections ((**A**)—CON, (**B**)—PMOS, magnification 20×); Quantitative analysis of myocardial collagen deposition (**C**). Bars represent means ± SEM; ** statistical significance at level *p* < 0.01 compared to the CON group. (*n* = 6 rats per group).

Representative photomicrographs of Picrosirius Red-stained myocardial sections from the experimental groups are presented in Figure 6. In the CON group, only minimal collagen deposition was observed within the myocardial tissue (Figure 6A). Conversely, the PMOS group exhibited markedly enhanced collagen accumulation, predominantly in the interstitial and perivascular regions (Figure 6B). Quantitative assessment of collagen deposition (Figure 6C) revealed significantly higher values (361%) in the PMOS group compared with the CON group (*p* < 0.01).

### 3.3. Impact of the LET + HFD on the Alterations of the Metabolic Indices, Hormone Levels, Oxidative Stress and Inflammatory Marker Levels

**Figure 7:**
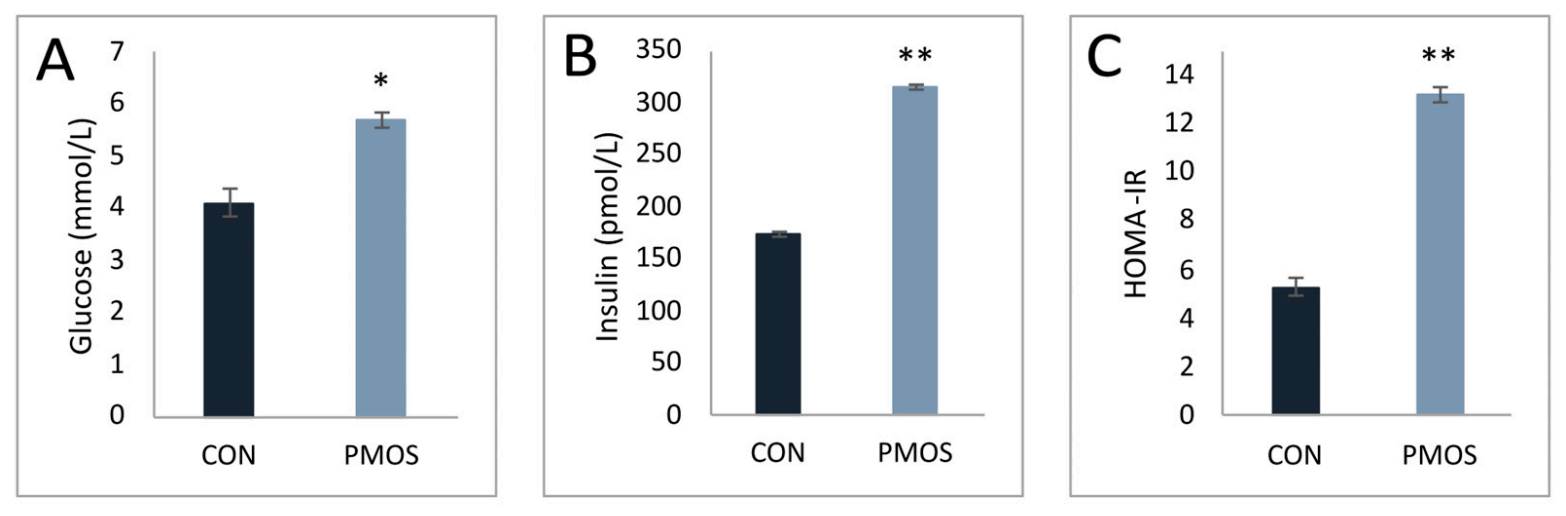
Basal glucose (**A**), insulin levels (**B**), and HOMA-IR (**C**). Bars represent means ± SEM; * Statistical significance at the level of *p* < 0.05 compared to CON group; ** Statistical significance at the level of *p* < 0.01 compared to CON group. (*n* = 6 rats per group).

Fasting whole-blood glucose was modestly but significantly higher in the PMOS group than in the CON group (Figure 7A; 35.98%, *p* < 0.05). In addition, significantly increased insulin levels (Figure 7B) (80.28%, *p* < 0.01) and HOMA-IR (Figure 7C) (147.81%, *p* < 0.01) suggest the presence of insulin resistance. Raw data are presented in Table S3.

**Figure 8:**
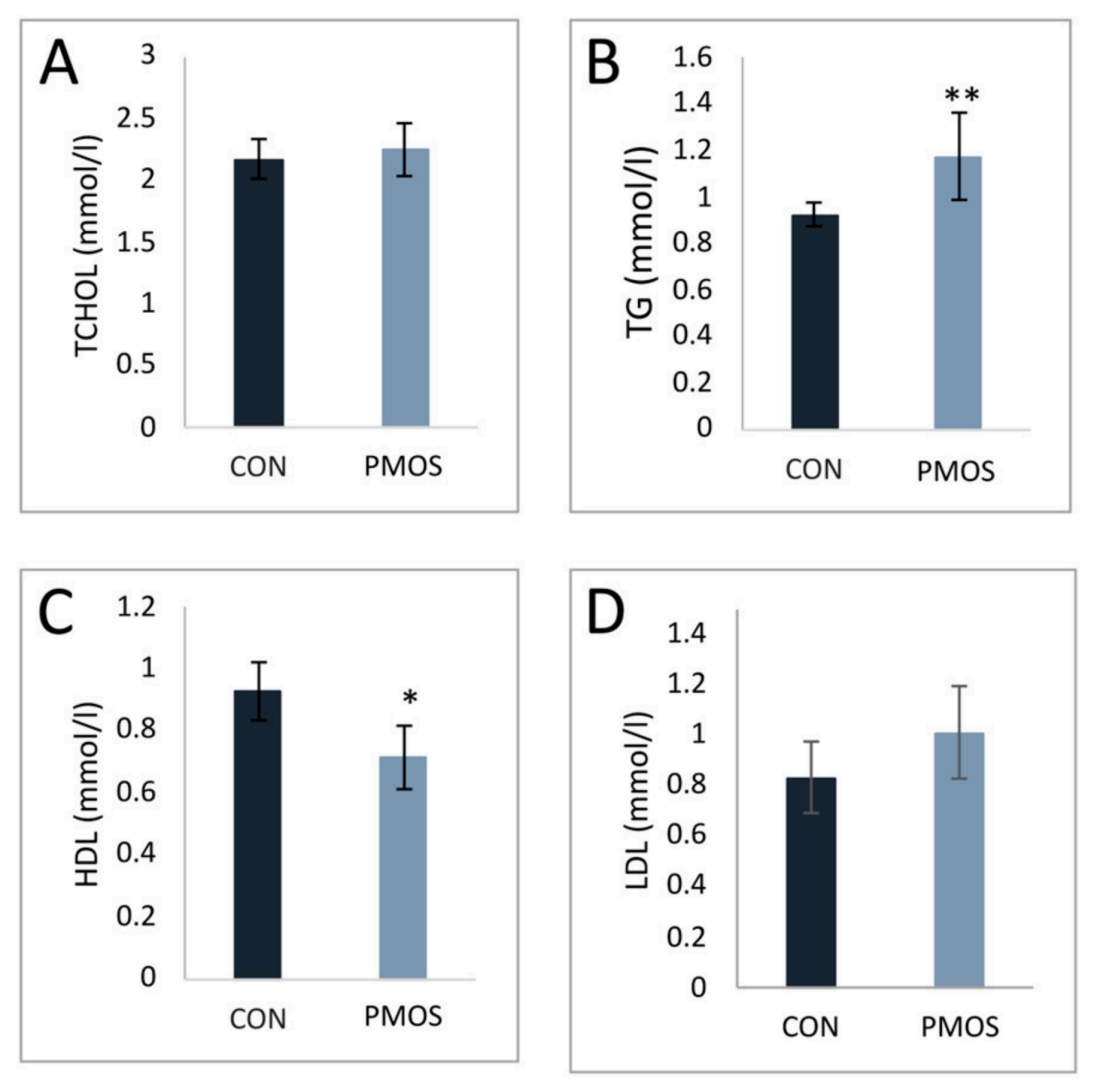
Levels of total cholesterol (**A**), triglycerides (**B**), high-density lipoproteins (**C**), and low-density lipoproteins (**D**). Bars represent means ± SEM; * Statistical significance at the level of *p* < 0.05 compared to CON group; ** Statistical significance at the level of *p* < 0.01 compared to CON group. (*n* = 6 rats per group).

Changes in the serum lipid profile are presented in Figure 8. Triglyceride levels (Figure 8B) were significantly elevated in the PMOS group (27.02%, *p* < 0.01), while HDL levels (Figure 8C) were significantly reduced in the experimental group (22.97%, *p* < 0.05). In addition, total cholesterol (Figure 8A) and LDL levels (Figure 8D) showed a slight tendency to increase in the PMOS group; however, these changes were not statistically significant.

**Figure 9:**
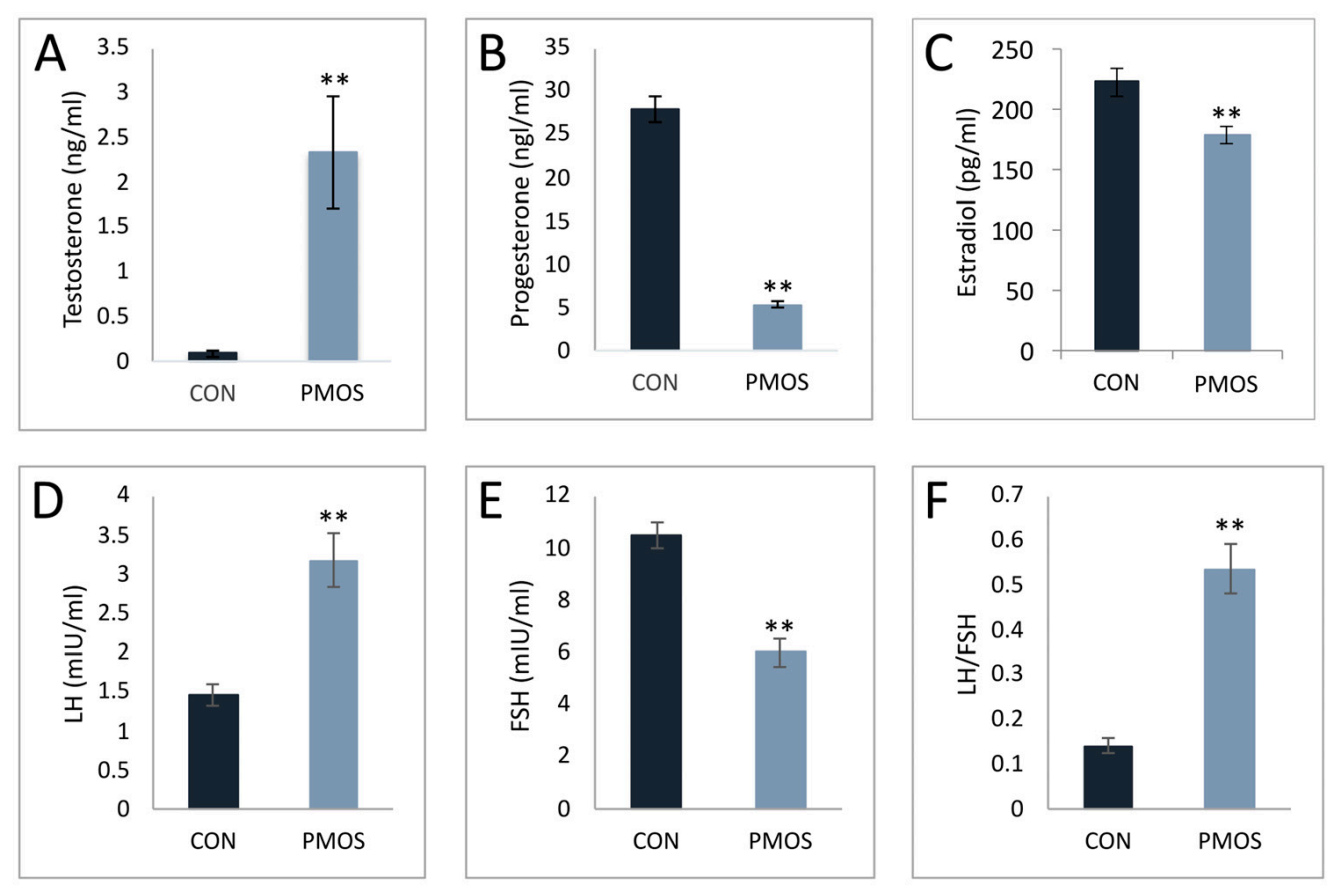
Levels of testosterone (**A**), progesterone (**B**), estradiol (**C**), LH (**D**), of FSH (**E**), and LH/FSH ratio (**F**). Bars represent means ± SEM; ** statistical significance at level *p* < 0.01 compared to the CON group. (*n* = 6 rats per group).

Serum concentrations of testosterone (Figure 9A) were significantly higher in the PMOS group compared to the CON group (2534.83%, *p* < 0.01), as were LH levels (Figure 9D) (111.14%, *p* < 0.01), whereas serum levels of estradiol (Figure 9C) (19.78%), progesterone (Figure 9B) (80.88%), and FSH (Figure 9E) (42.87%) were significantly lower in the PMOS group relative to the CON group (*p* < 0.01). Additionally, the LH/FSH ratio (Figure 9F) was significantly higher (270%, *p* < 0.01) in the PMOS group compared to the CON group. Raw data for LH, FSH and LH/FSH are presented in Table S3.

**Figure 10:**
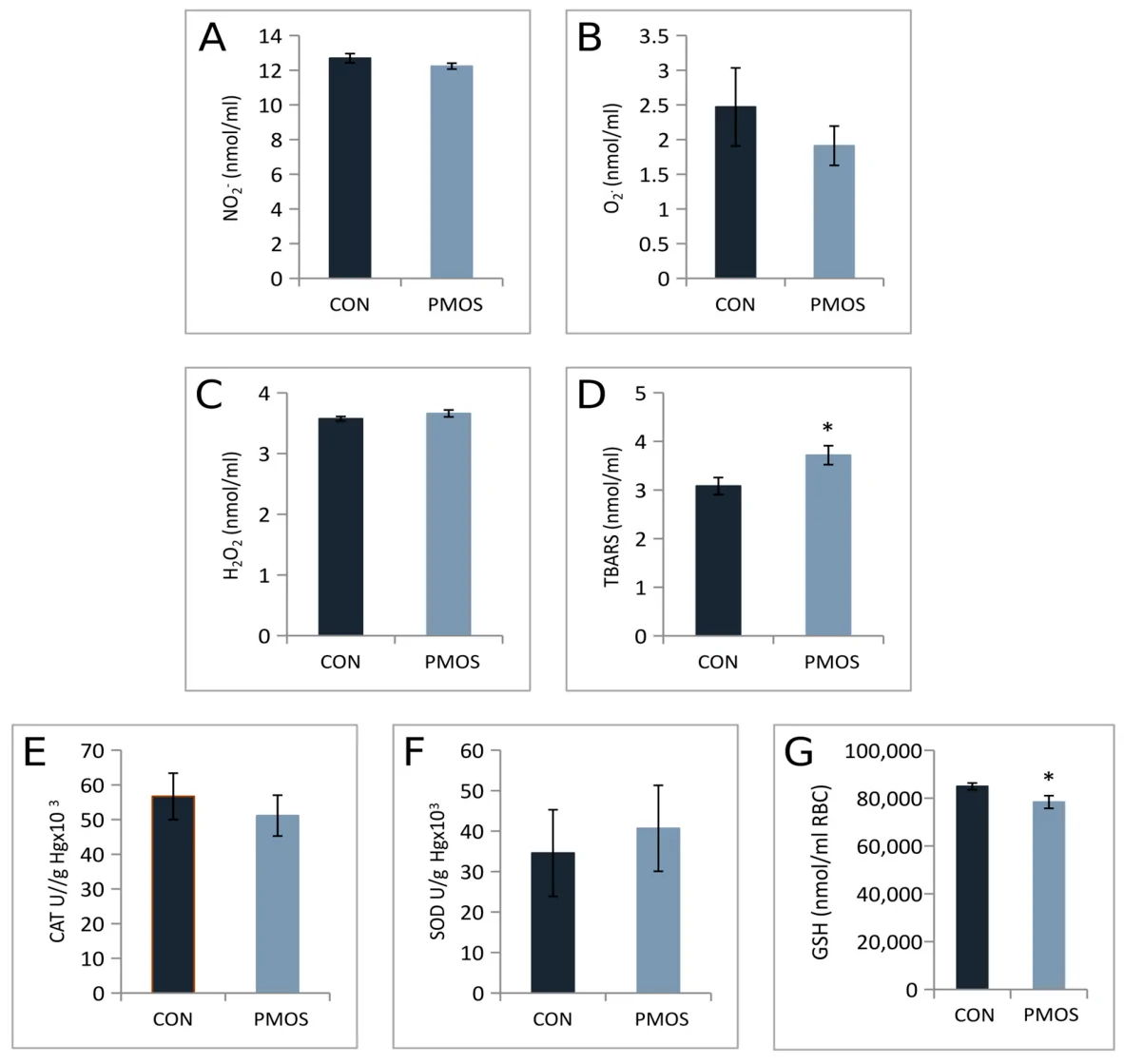
Oxidative stress parameters in the blood of rats. Levels of nitrites (NO_2_^−^) (**A**); levels of superoxide anion radical (O_2_^−^) (**B**); levels of hydrogen peroxide (H_2_O_2_) (**C**); index of lipid peroxidation (TBARS) (**D**); activity of catalase (CAT) (**E**); activity of superoxide dismutase (SOD) (**F**); level of reduced glutathione (GSH) (**G**). Bars represent means ± SEM; * Statistical significance at the level of *p* < 0.05 compared to the CON group. (*n* = 6 rats per group).

Index of lipid peroxidation, TBARS (Figure 10D) was significantly elevated (20.62%, *p* < 0.05) in the PMOS group compared to the CON g roup, whereas the other pro-oxidative parameters NO_2_^−^ (Figure 10A), O_2_^−^ (Figure 10B), and H_2_O_2_ (Figure 10C) remained unchanged. A significant reduction (7.70%, *p* < 0.05) in GSH (Figure 10G) was observed in the PMOS group compared with controls; however, no alterations were detected in the activities of SOD (Figure 10F) and CAT (Figure 10E).

**Figure 11:**
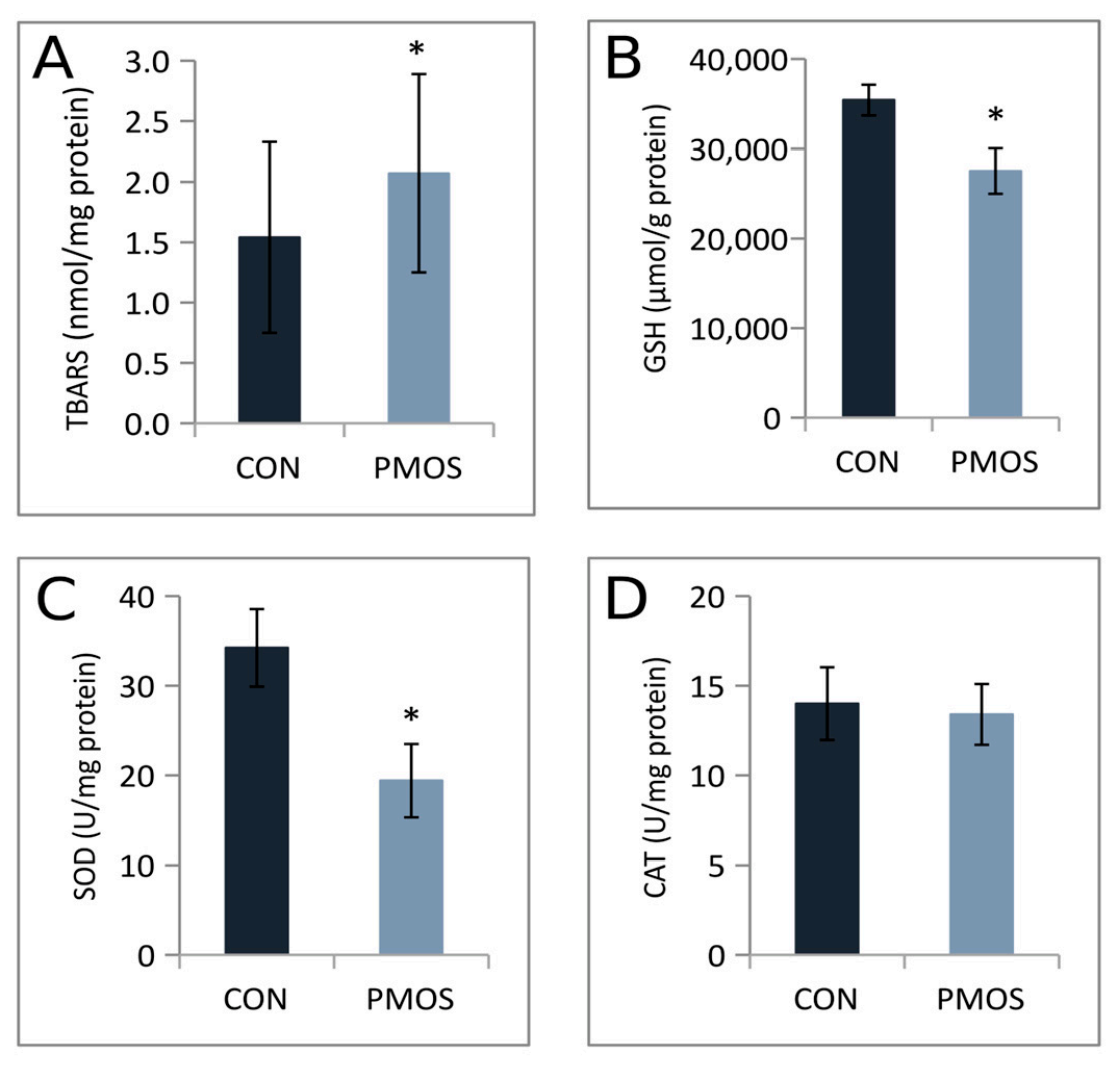
Oxidative stress parameters in ovarian tissue. Index of lipid peroxidation (TBARS) (**A**); level of reduced glutathione (GSH) (**B**); activity of superoxide dismutase (SOD) (**C**); activity of catalase (CAT) (**D**). Bars represent means ± SEM; * Statistical significance at the level of *p* < 0.05 compared to the CON group. (*n* = 6 rats per group).

Ovarian tissue oxidative stress parameters are presented in Figure 11. The index of lipid peroxidation, TBARS (Figure 11A), was significantly elevated (34.42%, *p* < 0.05) in the PMOS group compared with the CON group. In contrast, the levels of antioxidant parameters GSH (Figure 11B) and SOD activity (Figure 11C) were significantly reduced in the PMOS group (22.28%, *p* < 0.05 and (43.32%, *p* < 0.05), respectively). No significant changes were observed in CAT activity (Figure 11D) between the experimental groups.

**Figure 12:**
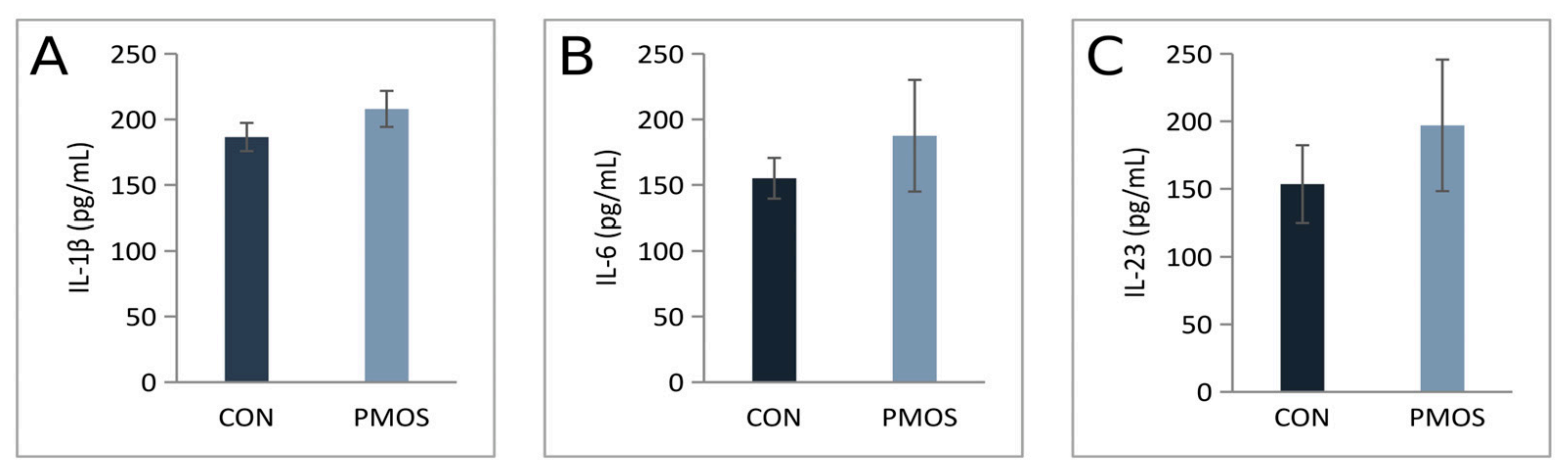
Levels of IL-1β (**A**); IL-6 (**B**); IL-23 (**C**); Bars represent means ± SEM. (*n* = 6 rats per group).

Serum levels of the inflammatory markers (Figure 12) IL-1β (Figure 12A), IL-6 (Figure 12B), and IL-23 (Figure 12C) showed no significant differences between the CON and PMOS groups.

## 4. Discussion

In our study, the combined LET + HFD protocol successfully induced a PMOS-like phenotype characterized by reproductive, endocrine, metabolic, oxidative, histomorphological, and cardiac alterations. Although there is no experimental model that fully recapitulates the pathophysiological mechanisms of human PMOS, LET + HFD protocol induced several key reproductive, endocrine, metabolic, and cardiometabolic features of PMOS, making it a valuable tool for investigating specific pathophysiological mechanisms (Figure S3) and evaluating potential therapeutic strategies. LET + HFD treatment increased body weight gain, while uterine weight was reduced compared with controls. Persistent diestrus was observed during estrous cycle monitoring, supporting successful induction of the PMOS-like phenotype. Reduced uterine weight was consistent with the hypoestrogenic environment resulting from aromatase inhibition and impaired estrogen-dependent trophic stimulation of the uterus. Similar changes in body weight have been reported in experimental PMOS models [25,26]. Notably, recent evidence has further demonstrated that significant body weight gain becomes evident as early as one week after LET + HFD induction and continues to increase during the following weeks, supporting the rapid development of the metabolic phenotype in this model [12], as observed in our study. However, the unchanged ovarian weight in our study suggests that ovarian enlargement may have occurred proportionally to overall body weight gain, which is consistent with some previous letrozole and LET + HFD models [27]. Increased body weight may reflect the metabolic component of the LET + HFD-induced phenotype, while reduced uterine mass may be related to disrupted folliculogenesis, androgen excess, estrogen deficiency, and altered steroid signaling, as described in previous studies [3,10,26,28].

Nowadays, PMOS is increasingly recognized as cardiometabolic disorder [8]. In this context, wavy muscle fibers, hypercellularity, cardiomyocyte enlargement, and increased collagen deposition observed in the LET + HFD group may reflect cardiac structural alterations associated with the combined endocrine and metabolic disturbances of this experimental phenotype. Previous studies have reported cardiorenometabolic disturbances and oxidative stress in letrozole-induced [29] as well as testosterone-induced [6] PMOS models, while other evidence suggests that letrozole exposure and androgen excess may be linked to cardiometabolic and structural alterations [30,31]. In our previous testosterone-induced PMOS model, cardiomyocyte cross-sectional area was increased (by 7.3%), whereas in the present LET + HFD model it was increased even more (by 15.5%) [6]. These findings suggest that the combination of hyperandrogenism and HFD may be associated with more pronounced cardiac morphometric alterations than androgen excess alone [32], although causality cannot be established from the present design. Picrosirius Red staining additionally revealed increased myocardial collagen deposition in the LET + HFD group, suggesting fibrotic remodeling-like changes. This is consistent with evidence that HFD-induced obesity can promote cardiac fibrosis and collagen accumulation [33]. Although androgen excess in PMOS is not directly comparable to supraphysiological androgen exposure, data from androgen-related cardiovascular studies support the concept that it may adversely affect cardiac structure [34]. The increased myocardial collagen deposition observed in the present study likely reflects the combined effects of endocrine and metabolic disturbances induced by the LET + HFD protocol. Hyperandrogenism, dyslipidemia, and metabolic dysfunction have been associated with extracellular matrix remodeling and the development of myocardial fibrosis in both experimental and clinical studies [35]. Furthermore, oxidative stress observed in our model, may activate profibrotic signaling pathways, thereby promoting excessive collagen deposition within the myocardium [36]. Although the underlying molecular mechanisms were not investigated in the present study, the increased collagen content detected by Sirius Red staining is consistent with early myocardial remodeling accompanying the cardiometabolic phenotype of the PMOS [37]. Therefore, the present findings support the relevance of the LET + HFD model for investigating structural cardiometabolic manifestations of PMOS-like phenotype, while further functional and molecular cardiac studies are needed.

Letrozole-induced aromatase inhibition leads to reduced estrogen synthesis and androgen accumulation [13,25]. Hyperandrogenemia is a common feature of letrozole-induced rodent models, including LET + HFD protocols [10,12,13,25,26,38,39]. Reduced estrogen feedback may contribute to increased GnRH/LH activity, while relatively low FSH impairs granulosa cell function and follicular maturation [25,38]. Nevertheless, hormonal responses in letrozole-induced models are not uniform, as some studies reported no significant changes in levels of hormones [40]. Such differences may reflect variations in dose, duration, strain, metabolic status, and timing of hormonal assessment.

Metabolic assessment suggested impaired glucose-insulin homeostasis and insulin resistance. However, because GTT and ITT were not performed, these findings should be interpreted cautiously and cannot fully define functional insulin resistance. Previous studies indicate that LET + HFD protocols reproduce both reproductive and metabolic components of PMOS-like phenotype [12,26,38,39,41]. Letrozole primarily promotes hyperandrogenism, while HFD-related adipose tissue expansion and hyperinsulinemia may further aggravate endocrine dysregulation [26,38]. Insulin resistance is closely linked to ovarian steroidogenesis, as hyperinsulinemia can stimulate androgen production in theca cells, acting synergistically with LH, reduce SHBG synthesis, and increase androgen bioavailability [10,25,26,38,41]. These processes may contribute to reduced estradiol and progesterone synthesis, impaired granulosa cell function, and disrupted folliculogenesis [25,26,41]. These metabolic disturbances result from the complex interplay between androgen excess, impaired insulin signaling, adipose tissue dysfunction, and reduced estrogen availability secondary to aromatase inhibition. Consequently, in the LET + HFD model, hyperandrogenism acts synergistically with dietary fat exposure to promote metabolic dysfunction, consistent with both clinical observations and previous experimental studies [42,43].

Histological findings supported the endocrine and metabolic data. Ovarian sections showed reduced developing follicles, numerous atretic and cystic follicles, and fewer corpora lutea, consistent with previous reports [10,13,25,30,38]. These changes may be related to increased LH/FSH ratio, androgen excess, impaired granulosa cell function, and follicular arrest [25,38]. Estrogen deficiency may further promote granulosa cell apoptosis and thinning of the follicular wall [38], while HFD-associated metabolic stress may potentiate androgen synthesis and cyst formation [44]. Uterine tissue changes, including thinning of luminal and glandular epithelium, stromal layer, and endometrium, are also consistent with hypoestrogenism and endocrine imbalance. These findings agree with previous reports showing uterine alterations in letrozole-induced PMOS-like models, particularly when combined with HFD [30,45].

The enlargement of subcutaneous and periovarian adipocytes further supports the presence of metabolic dysregulation in the LET + HFD group. Previous study demonstrated impaired insulin signaling in letrozole-induced PMOS model [46]. The addition of HFD has been shown to aggravate metabolic dysfunction and insulin resistance, making the model more representative of obese, insulin-resistant PMOS-like phenotype [12]. Therefore, increased adipocyte area and diameter in our study may represent morphological manifestations of adipose tissue expansion and metabolic disturbance. Hyperandrogenism and hyperinsulinemia are known to disturb lipid metabolism and may contribute to elevated triglycerides, altered hepatic lipid handling, and reduced HDL [25,38], as observed in our study. However, lipid alterations in experimental PMOS models are heterogeneous. Some studies reported selective triglyceride elevation, while others showed increased total cholesterol and LDL without HDL changes [32]. Such variability may depend on diet composition, protocol duration, severity of insulin resistance, and model characteristics [37,47]. Moreover, women with PMOS exhibit increased visceral adiposity and metabolic disturbances irrespective of obesity status [48]. Similarly, visceral adipocytes in our study showed significantly increased diameter and area, supporting the relevance of the LET + HFD-induced PMOS-like model.

Increased visceral adiposity may contribute to oxidative stress and metabolic dysfunction associated with PMOS-like phenotype [49]. Hyperandrogenism, altered glucose-insulin metabolism, and dyslipidemia may contribute to ROS generation and redox imbalance [39]. Moreover, in our study, oxidative stress analysis showed lipid peroxidation and reduced GSH suggests that antioxidant defenses may be insufficient to fully counteract lipid oxidative damage [39]. Ovarian tissue analysis further strengthened these findings, showing elevated TBARS with reduced GSH and SOD, while CAT activity was relatively preserved, supporting the concept that ovarian oxidative stress may be more evident than peripheral redox imbalance in experimental PMOS-like phenotype. Overall, these findings are consistent with previous studies reporting increased pro-oxidant production and impaired antioxidant defense in PMOS models and related reproductive tissues [10,25,50,51]. In contrast, IL-6, IL-1β, and IL-23 did not differ significantly between groups. Therefore, the present data do not support marked systemic inflammatory activation under the applied experimental conditions. Previous studies using letrozole-induced models reported variable inflammatory responses, depending on protocol duration, diet, and severity of metabolic impairment [10]. The absence of significant cytokine changes may reflect either a limited inflammatory response or insufficient power to detect subtle differences due to the small sample size. Oxidative stress may occur independently of detectable systemic cytokine changes, as inflammatory activation requires additional redox-sensitive signaling mechanisms such as NF-κB [52]. Thus, while the LET + HFD model reproduced metabolic and oxidative features of PMOS-like phenotype, systemic inflammatory alterations may depend on additional experimental and biological factors.

## 5. Study Limitations and Future Directions

Several limitations should be acknowledged. Although the sample size was supported by an a priori power analysis and complied with the Reduction principle of the 3Rs, the relatively small number of animals per group warrants cautious interpretation of modest or non-significant findings. The absence of letrozole-only and high-fat diet-only groups precludes discrimination of the individual contributions. Nevertheless, the principal objective of the present study was to comprehensively characterize an established LET + HFD-induced PMOS-like model rather than to investigate the independent effects of each intervention. In addition, glucose tolerance and insulin tolerance tests were not performed, and food intake and adipose tissue mass were not assessed, limiting the functional assessment of glucose metabolism, insulin sensitivity and comprehensive metabolic characterization of the model. Likewise, cardiac alterations were evaluated primarily at the structural level, without functional or molecular characterization of myocardial remodeling. Therefore, the present findings should be considered exploratory and require confirmation in larger mechanistic and translational studies. Future research should incorporate dynamic metabolic testing, functional cardiovascular assessment, molecular analyses of myocardial remodeling and fibrosis, and additional experimental groups to further dissect the individual and combined effects of letrozole and high-fat diet, as well as to facilitate translation of these findings to women with PMOS.

## 6. Conclusions

The present study provides a comprehensive reproductive and cardiometabolic characterization of a letrozole- and high-fat-diet-induced PMOS-like rat model. Beyond the established reproductive and endocrine abnormalities, the model exhibited metabolic, oxidative, and histomorphological alterations involving multiple organs, including the heart, supporting its utility for investigating the multisystem pathophysiology of PMOS. Future mechanistic, functional, and translational studies are needed to further elucidate the biological mechanisms and clinical relevance of these findings.

## Figures and Tables

**Table 1:** Changes in ovary, uterus and heart expressed to 100 g of BW. Data are presented as mean ± SEM.** statistical significance at level *p* < 0.01 compared to the CON group. (*n* = 6 rats per group).

Groups	Ovarian Weight (mg/ 100 g BW)	1 cm of Uterus Weight (mg/ 100 g BW)	Heart Weight (mg/ 100 g BW)
CON	0.014 ± 0.001	0.017 ± 0.003	0.304 ± 0.019
PMOS	0.017 ± 0.002	0.007 ± 0.002 **	0.313 ± 0.010

## Data Availability

The raw data supporting the conclusions of this article will be made available by the authors on request.

## Supplementary Materials

The following supporting information can be downloaded at: https://www.mdpi.com/article/10.3390/pathophysiology33030061/s1, Figure S1: Experimental design and timeline of the study; Figure S2: Representative gross morphology of ovaries from control (left) and LET+HFD-induced PMOS (right) rats; Figure S3: Proposed mechanisms underlying the PMOS phenotype induced by letrozole combined with a high-fat diet; Table S1: Descriptive statistics of the biochemical parameters; Table S2: Individual estrous-cycle patterns recorded during the final 12 days of the experimental protocol; Table S3: Individual raw data for fasting glucose, fasting insulin, LH, FSH, HOMA-IR, and the LH/FSH ratio.

## Abbreviations

The following abbreviations are used in this manuscript:

PMOS	Polyendocrine metabolic ovarian syndrome
CON	control
HPO	hypothalamic–pituitary–ovarian
GnRH	Gonadotropin-Releasing Hormone
FSH	Follicle-Stimulating Hormone
LH	Luteinizing Hormone
HFD	high-fat diet
HOMA-IR	Homeostatic Model Assessment of Insulin Resistance
H/E	hematoxylin and eosin staining
TCHOL	total cholesterol
TG	triglycerides
LDL	low-density lipoproteins
HDL	high-density lipoproteins
T	total testosterone
P4	progesterone
E2	estradiol
TBARS	thiobarbituric acid reactive substances
NO_2_^−^	nitrites
H_2_O_2_	hydrogen peroxide
O_2_^−^	superoxide anion radical
SOD	superoxide dismutase
GSH	reduced glutathione
CAT	catalase
