# Supplementary material for: Reproductive and Cardiometabolic Characterization of a Letrozole- and High-Fat Diet-Induced PMOS-like Rat Model: An Experimental Study
Source: Pathophysiology. 2026 Aug 11;33(3):61. doi: 10.3390/pathophysiology33030061 (PMC13510558; doi:10.3390/pathophysiology33030061)
Supplement: Supplementary file 1 [file pathophysiology-33-00061-s001.zip › pathophysiology-4418857-supplementary.pdf]

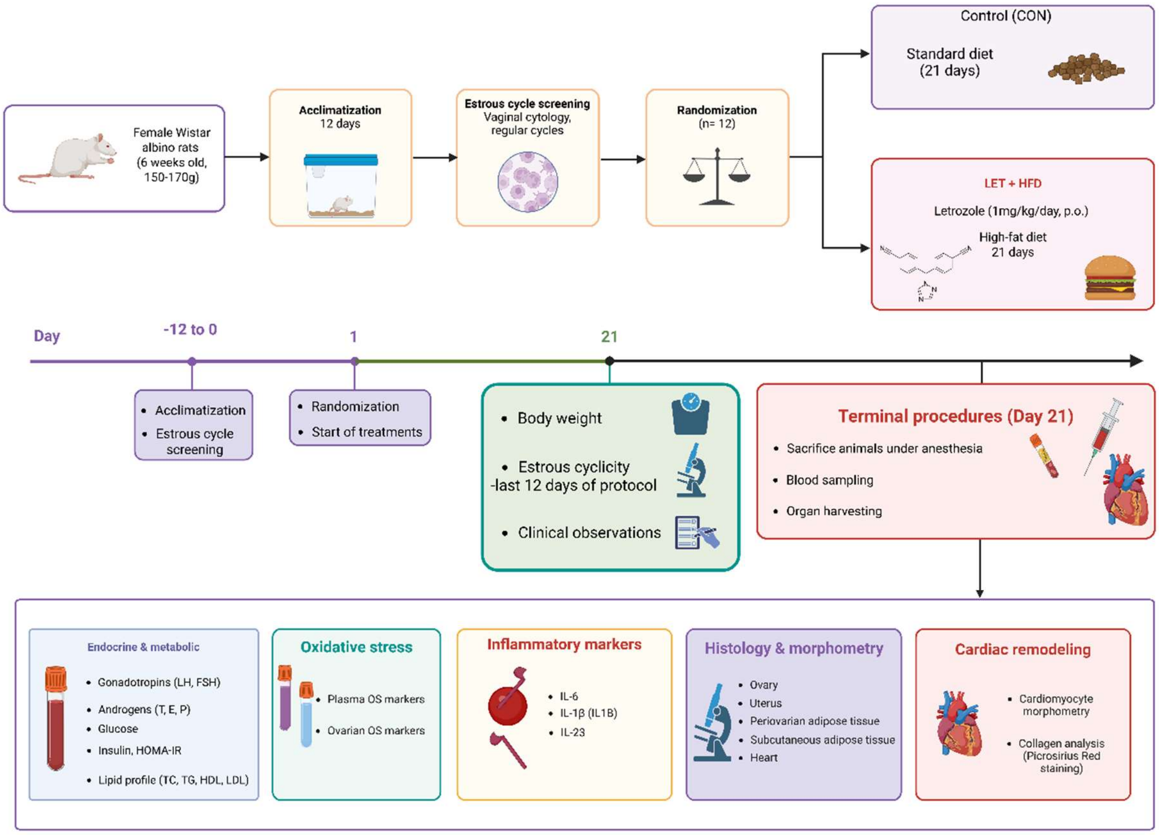

Figure S1. Experimental design and timeline of the study. Abbreviations: CON, control; LET, letrozole; HFD, high-fat diet; p.o., oral administration; LH, luteinizing hormone; FSH, follicle-stimulating hormone; T, testosterone; E, estradiol; P, progesterone; HOMA-IR, homeostatic model assessment of insulin resistance; TC, total cholesterol; TG, triglycerides; HDL, high-density lipoprotein cholesterol; LDL, low-density lipoprotein cholesterol; OS, oxidative stress; IL, interleukin.

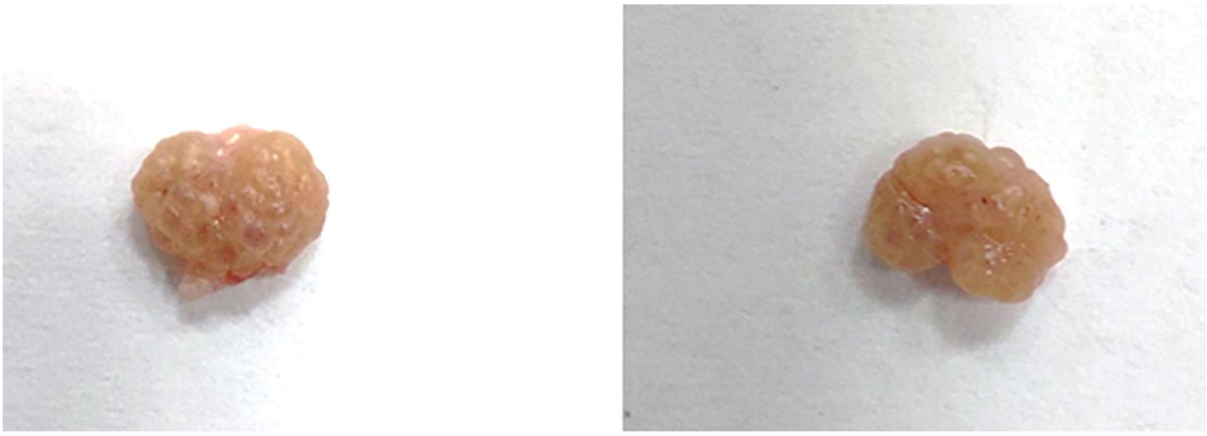

Figure S2. Representative gross morphology of ovaries from control (left) and LET+HFD-induced PMOS (right) rats. The control ovary exhibits a compact, smooth, and regularly contoured appearance with a light-red coloration and multiple visible corpora lutea. In contrast, the PMOS ovary appears slightly enlarged, paler, and more irregular, with a multilobulated, nodular surface and several rounded translucent protrusions suggestive of underlying cystic follicles, consistent with the histopathological findings presented in the main manuscript.

https://doi.org/10.3390/pathophysiology33030061

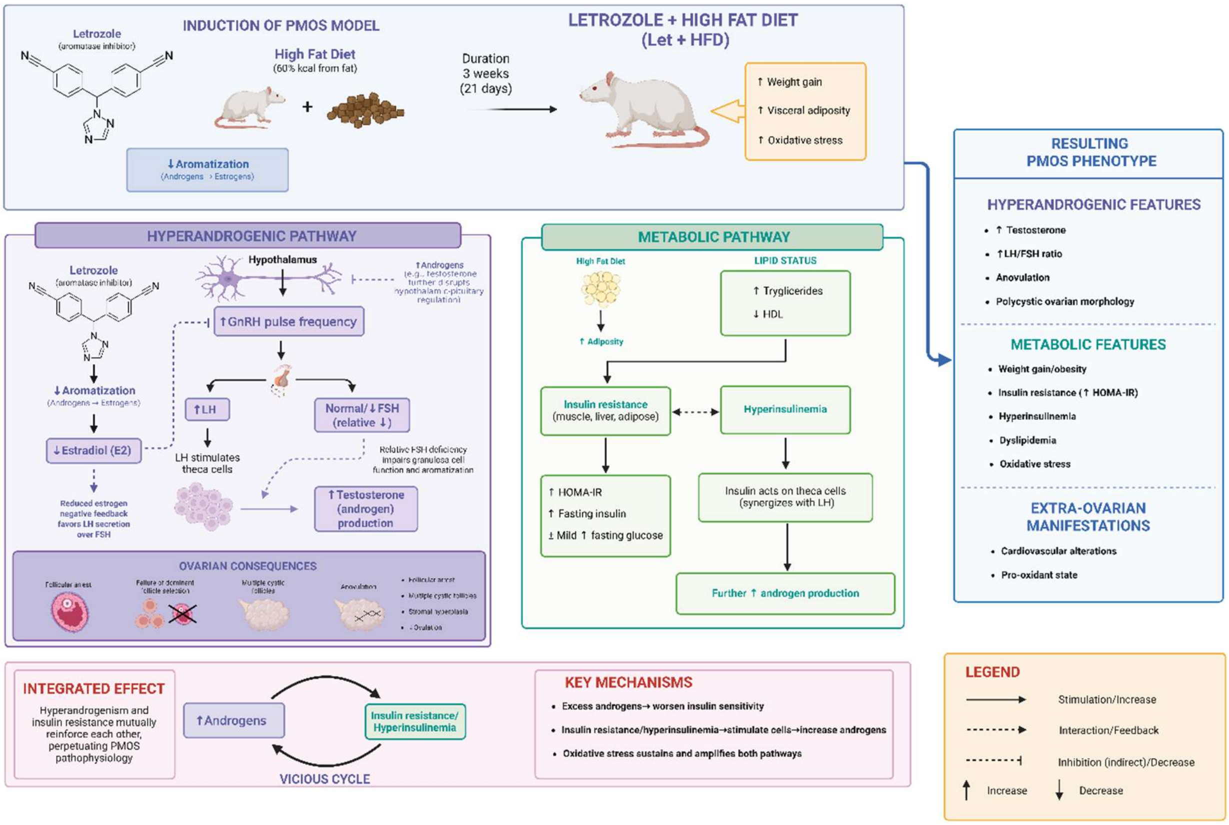

Figure S3. Proposed mechanisms underlying the PMOS phenotype induced by letrozole combined with a high-fat diet. The schematic illustrates the complementary hyperandrogenic and metabolic pathways through which combined letrozole (LET) administration and high-fat diet (HFD) exposure may induce a PMOS-like phenotype in female rats. Letrozole-mediated aromatase inhibition reduces the conversion of androgens to estrogens, resulting in decreased estradiol levels and disruption of hypothalamic–pituitary–ovarian feedback. The consequent increase in gonadotropin-releasing hormone pulse frequency, relative predominance of luteinizing hormone over follicle-stimulating hormone, and stimulation of ovarian theca cells promote androgen production, follicular arrest, anovulation, and polycystic ovarian morphology. In parallel, HFD-induced adiposity and dyslipidemia contribute to insulin resistance and compensatory hyperinsulinemia. Insulin may further enhance luteinizing hormone-stimulated androgen synthesis in theca cells. Hyperandrogenism and insulin resistance therefore mutually reinforce one another, establishing a vicious cycle that contributes to the development and maintenance of the reproductive, metabolic, oxidative, and extra-ovarian manifestations of the model. Abbreviations: LET, letrozole; HFD, high-fat diet; GnRH, gonadotropin-releasing hormone; LH, luteinizing hormone; FSH, follicle-stimulating hormone; E2, estradiol; HOMA-IR, homeostatic model assessment of insulin resistance; HDL, high-density lipoprotein cholesterol; PMOS, polyendocrine metabolic ovary syndrome.

https://doi.org/10.3390/pathophysiology33030061

Table S1. Descriptive statistics of the biochemical parameters. Data are presented as mean ± SD for normally distributed variables and as median (IQR) for non-normally distributed variables (n=6).

Parameter	Descriptive statistics	
	**CON**	**PMOS**
Glucose	4.1±0.64	5.68±0.33
Insulin	174.17(12.3)	313.94(12.21)
HOMA-IR	5.09(2.44)	12.81(1.78)
Total cholesterol	2.16±0.29	2.25±0.16
Triglycerides	0.92±0.09	1.17±0.14
HDL	0.92±0.17	0.71±0.16
LDL	0.82±0.34	1.01±0.45
Testosterone	0.11±0.08	2.34±0.97
Estradiol	237.8±20.32	180.33±19.02
Progesterone	28(4)	5.49(0.69)
LH	1.47±0.33	3.17±0.84
FSH	10.51±1.17	6.00±1.31
LH/FSH	0.14±0.04	0.53±0.13
Plasma NO_2^-	12.85±0.58	12.23±0.34
Plasma O_2^-	2.51±0.98	1.92±0.57
Plasma H_2O_2	3.52(0.14)	3.64(0.12)
Plasma TBARS	3.08±0.4	3.71±0.33
Lysate CAT	54.55±12.56	51.15±11.76
Lysate SOD	34.58±18.54	40.7±21.23
Lysate GSH	84935.96±3192.06	78439.48±4173.19
Ovarian TBARS	1.5±0.43	2.07±0.37
Ovarian GSH	35672.18±3033.16	27524.77±5056.79
Ovarian SOD	33.37±7.73	19.41±8.2
Ovarian CAT	14±4.54	13.4±3.43
IL-1B	178.04±52.3	208.07±94.74
Il-6	106.46±13.61	187.34±50.8
Il-23	146.94±26.83	196.38±38.08

https://doi.org/10.3390/pathophysiology33030061

Table S2. Individual estrous-cycle patterns recorded during the final 12 days of the experimental protocol. Each row represents one animal. P, proestrus; E, estrus; M, metestrus; D, diestrus. Control animals exhibited regular cyclic changes with inter-individual variation in cycle timing, whereas all LET+HFD-induced PMOS-like animals remained in persistent diestrus throughout the observation period.

	**Day 10**	**Day 11**	**Day 12**	**Day 13**	**Day 14**	**Day 15**	**Day 16**	**Day 17**	**Day 18**	**Day 19**	**Day 20**	**Day 21**
CON 1	P	E	M	D	P	E	M	D	P	E	M	D
CON 2	D	P	E	M	D	P	E	M	D	P	E	M
CON 3	M	D	D	P	E	M	D	P	E	M	D	P
CON 4	E	M	D	P	E	M	D	P	E	M	D	P
CON 5	P	E	D	D	P	E	M	D	P	E	M	D
CON 6	D	P	E	M	D	D	P	E	M	D	P	E
PMOS 1	D	D	D	D	D	D	D	D	D	D	D	D
PMOS 2	D	D	D	D	D	D	D	D	D	D	D	D
PMOS 3	D	D	D	D	D	D	D	D	D	D	D	D
PMOS 4	D	D	D	D	D	D	D	D	D	D	D	D
PMOS 5	D	D	D	D	D	D	D	D	D	D	D	D
PMOS 6	D	D	D	D	D	D	D	D	D	D	D	D

https://doi.org/10.3390/pathophysiology33030061

Table S3. Individual raw data for fasting glucose, fasting insulin, LH, FSH, HOMA-IR, and the LH/FSH ratio.

**Parameter**	**Glucose (mmol/L)**		**INS (pmol/L)**		**HOMA-IR**	
Groups	CON	PMOS	CON	PMOS	CON	PMOS
Rat 1	4.1	6.2	174.78	311.97	5.30	14.32
Rat 2	3.8	5.4	173.55	315.91	4.88	12.63
Rat 3	3.3	5.5	182.64	319.25	4.46	13.00
Rat 4	3.9	5.5	166.28	307.95	4.80	12.54
Rat 5	5.2	5.5	179.36	306.85	6.901	12.50
Rat 6	4.3	6	168.41	321.78	5.36	14.30
Parameter	LH (mIU/ml)		FSH (mIU/ml)		LH/FSH	
Groups	CON	PMOS	CON	PMOS	CON	PMOS
Rat 1	1.72	4.25	9.79	6.22	0.17	0.68
Rat 2	1.78	2.52	11.64	5.79	0.15	0.43
Rat 3	1.35	2.68	10.52	7.24	0.12	0.37
Rat 4	1.82	3.46	9.14	4.92	0.19	0.70
Rat 5	1.05	2.18	9.84	4.24	0.10	0.51
Rat 6	1.15	3.96	12.16	7.64	0.09	0.51

https://doi.org/10.3390/pathophysiology33030061
